# A deep learning approach to predict collateral flow in stroke patients using radiomic features from perfusion images

**DOI:** 10.3389/fneur.2023.1039693

**Published:** 2023-02-21

**Authors:** Giles Tetteh, Fernando Navarro, Raphael Meier, Johannes Kaesmacher, Johannes C. Paetzold, Jan S. Kirschke, Claus Zimmer, Roland Wiest, Bjoern H. Menze

**Affiliations:** ^1^Department of Computer Science, Technische Universität München, München, Germany; ^2^Neuroradiology, Klinikum Rechts der Isar, Technische Universität München, München, Germany; ^3^Institute for Diagnostic and Interventional Neuroradiology, Inselspital University Hospital, Bern, Switzerland; ^4^Department of Quantitative Biomedicine, University of Zurich, Zurich, Switzerland

**Keywords:** collateral flow, radiomics, perfusion, reinforcement learning, image descriptors, angiography, auto-encoder, deep learning

## Abstract

Collateral circulation results from specialized anastomotic channels which are capable of providing oxygenated blood to regions with compromised blood flow caused by arterial obstruction. The quality of collateral circulation has been established as a key factor in determining the likelihood of a favorable clinical outcome and goes a long way to determining the choice of a stroke care model. Though many imaging and grading methods exist for quantifying collateral blood flow, the actual grading is mostly done through manual inspection. This approach is associated with a number of challenges. First, it is time-consuming. Second, there is a high tendency for bias and inconsistency in the final grade assigned to a patient depending on the experience level of the clinician. We present a multi-stage deep learning approach to predict collateral flow grading in stroke patients based on radiomic features extracted from MR perfusion data. First, we formulate a region of interest detection task as a reinforcement learning problem and train a deep learning network to automatically detect the occluded region within the 3D MR perfusion volumes. Second, we extract radiomic features from the obtained region of interest through local image descriptors and denoising auto-encoders. Finally, we apply a convolutional neural network and other machine learning classifiers to the extracted radiomic features to automatically predict the collateral flow grading of the given patient volume as one of three severity classes - no flow (0), moderate flow (1), and good flow (2). Results from our experiments show an overall accuracy of 72% in the three-class prediction task. With an inter-observer agreement of 16% and a maximum intra-observer agreement of 74% in a similar experiment, our automated deep learning approach demonstrates a performance comparable to expert grading, is faster than visual inspection, and eliminates the problem of grading bias.

## 1. Introduction

Collateral circulation results from specialized anastomotic channels which are present in most tissues and capable of providing nutrient perfusion to regions with compromised blood flow due to ischemic injuries caused by ischemic stroke, coronary atherosclerosis, peripheral artery disease, and similar conditions or diseases (1). Collateral circulation helps to sustain blood flow in the ischaemic areas in acute, subacute, or chronic phases after an ischaemic stroke or transient ischaemic attack (2). The quality of collateral circulation has been convincingly established as a key factor in determining the likelihood of successful reperfusion and favorable clinical outcome (3). It is also seen as one of the major determinants of infarct growth in the early time windows which is likely to have an impact on the chosen stroke care model that is the decision to transport or treat eligible patients immediately.

A high number of imaging methods exist to assess the structure of the cerebral collateral circulation and several grading criteria have been proposed to quantify the characteristics of collateral blood flow. However, this grading is mostly done through visual inspection of the acquired images which introduces two main challenges.

First, there are *biases and inconsistencies in the current grading approaches*: There is a high tendency of introducing bias in the final grade assigned to a patient depending on the experience level of the clinician. There are inconsistencies also in the grade assigned by a particular clinician at different times for the same patient. These inconsistencies are quantified at 16% interobserver agreement and a maximum intraobserver agreement of 74% respectively in a similar study by Ben Hassen et al. (4).

Second, *grading is time-consuming and tedious*: Aside the problem of bias prediction, it also takes the clinician several minutes to go through the patient images to first select the correct image sequence, detect the region of collateral flow and then to be able to assign a grading a period of time which otherwise could have been invested in the treatment of the patient.

In this work, we analyze several machine learning and deep learning strategies that aim toward automating the process of collateral circulation grading. We present a set of solutions focusing on two main aspects of the task at hand.

First, *the region of interest needs to be identified*. We automate the extraction of the region of interest (ROI) from the patient images using deep reinforcement learning (RL). This is necessary for achieving a fully automated system that will require no human interaction and save the clinician the time spent on performing this task.

Finally, *the region of interest needs to be processed and classified*. We consider various feature extraction schemes and classifiers suitable for the task described above. This helps to extract useful image features, both learned and hand-crafted, which are relevant to the classification task. We predict digitally subtracted angiography (DSA) based collateral flow grading from MR perfusion images in this task. This saves the time required in choosing the right DSA sequence from the multiple DSA sequences acquired and helps achieve a fully automated system.

### 1.1. Prior work and open challenges

#### 1.1.1. Imaging criteria for cerebral collateral circulation

Imaging methods for assessing cerebral collateral flow can be grouped under two main classification schemes, invasive vs. non-invasive and structural vs. functional imaging. Structural imaging methods provide information about the underlying structure of the cerebral collateral circulation network. Some of the commonly used structural imaging modalities are traditional single-phase computed tomography angiography (CTA), time-of-flight magnetic resonance angiography (TOF-MRA), and digitally subtracted angiography (DSA), among others. Other imaging modalities have been used in clinical practice and relevant research areas in accessing the structure of the cerebral collateral circulation are discussed in Liu et al. (2), McVerry et al. (5), Martinon et al. (6). DSA is the gold standard for assessing the collateral flow, however, due to the associated high cost and invasive nature, other non-invasive methods like CTA and MRA are commonly used (2).

Functional imaging methods are used to assess the function of the underlying cerebral collateral circulation. Single-photon emission CT (SPECT), MR perfusion, and positron emission tomography (PET) are examples of imaging methods that provide functional information about the cerebral collateral flow. MR perfusion imaging is often followed by a post-processing step to extract parametric information. Very common parametric information includes the *time-to-peak* (T_*max*_) time taken for the blood flow to reach its peak (max) at a given region in the brain, *relative blood flow* (rBF) volume of blood flowing through a given brain tissue per unit of time, and *relative blood volume* (rBV) volume of blood in a given brain tissue relative to an internal control (e.g. normal white matter or an arterial input function). Functional imaging is sometimes combined with structural imaging either in a single scanning procedure or separate procedures and can serve as complementing information in the decision making process. Here, structural imaging is oftentimes used to map the anatomy and probe tissue microstructure.

MRI perfusion and diffusion have evolved as key biomarkers in determining collateralization of stroke patients, and a patient stratification based on these markers has been proposed repeatedly (7). At the same time, a qualitative CTA and DSA based grading are the most common approaches for evaluating collateralization (8–10).

#### 1.1.2. Cerebral collateral flow grading

Cerebral collateral circulation plays an important role in stabilizing cerebral blood flow when the normal blood circulation system is compromised in cases of acute, subacute, or chronic ischaemic stroke. The quality of the cerebral collateral circulation system is one of the factors that determine the speed of infarct growth and the outcome of stroke treatment and reperfusion therapies. A poor collateral flow is associated with worse outcomes and faster growth of infarcts while a good collateral flow is associated with good outcomes and slower growth of infarcts in acute stroke treatment (11). Due to the important role played by cerebral collateral blood flow, various grading scales and their association with risk factors and treatment outcomes have been discussed extensively in literature.

Several grading systems have been proposed for assessing the quality of the collateral circulation network. Among these grading systems, the DSA based system proposed by the American Society of Interventional and Therapeutic Neuroradiology/Society of Interventional Radiology (ASITN/SIR) is the most widely accepted scheme. This grading system describes the collateral flow as one of five levels of flow which are; absence of collaterals (0), slow collaterals (1), rapid collaterals (2), partial collaterals (3), and complete collaterals (4) to the periphery of the ischaemic site (2, 12). In most studies that use the ASITN/SIR scheme, the grading scale is merged into three levels—grades 0–1 (poor), 2 (moderate) and 3–4 (good collateral) flow. CTA based systems also have several grading schemes ranging from two (good, bad) to five (absent, diminished >50%, <50%, equal, more) labels (12).

The relationship between pretreatment collateral grade and vascular recanalization has been assessed for patients who received endovascular therapy for acute cerebral ischemia from two distinct study populations by Bang et al. (13). The study showed that 14.1, 25.2, and 41.5% of patients with poor, good, and excellent pretreatment collaterals respectively achieved complete revascularization. Another study by Bang et al. (14) on the relationship between MRI diffusion and perfusion lesion indices, angiographic collateral grade, and infarct growth showed that the greatest infarct growth occurred in patients with both non-recanalization and poor collaterals. Mansour (15) assessed collateral pathways in acute ischemic stroke using a new grading scale (Mansour Scale) and correlated the findings with different risk factors, clinical outcomes, and recanalization rates with endovascular management. More research (13–17) has been conducted into the relationship between the cerebral collateral circulation, its grading, and the clinical outcome of the choice of treatment of acute ischemic stroke, and they all confirm a positive association between collateral flow and the success of the outcome.

Due to the crucial role played by collateral circulation, it is a common practice in most clinical procedures to determine the quality of a patient's collateral as first-hand information toward the choice of the treatment or care model. This grading is done manually by inspecting patient scans which is time-consuming and also introduces some level of bias in the final grade assigned to a patient. Ben Hassen et al. (4) evaluated the inter-and intraobserver agreement in angiographic leptomeningeal collateral flow assessment on the ASITN/SIR scale and found an overall interobserver agreement κ = 0.16 ± 6.5 × 10^−3^ among 19 observers with grades 0 and 1 being associated with the best results of κ = 0.52 ± 0.001 and κ = 0.43 ± 0.004 respectively. By merging the scales into two classes, poor collaterals (grade 0, 1, or 2), versus good collaterals (grade 3 or 4), the interobserver agreement increased to κ = 0.27 ± 0.014. The same study recorded maximum intraobserver agreements of κ = 0.74 ± 0.1 and κ = 0.79 ± 0.11 for the ASITN/SIR and dichotomized scales respectively. McHugh (18) presented a study on interrater reliability and the kappa statistic as a measure of agreement and recommended a moderate interobserver agreement of 0.60 ≤ κ ≤ 0.79 as a minimum requirement for medical data and study. These results are evidence of the need to automate the collateral grading process to achieve speed and consistency in the assigned grading.

Methods for automating the grading of collateral flow have not yet been properly explored in literature. Kersten-Oertel et al. (19) presented an automated technique to compute a collateral circulation score based on differences seen in mean intensities between left and right cerebral hemispheres in 4D angiography images and found a good correlation between the computed score and radiologist score (*r*^2^ = 0.71) and good separation between good and intermediate/poor groups. Grunwald et al. (20) used a machine learning approach to categorize the degree of collateral flow in 98 patients who were eligible for mechanical thrombectomy and generated an e-CTA collateral score (CTA-CS) for each patient. The experiments showed that the e-CTA generated improved the intraclass correlation coefficient between three experienced neuroradiologists from 0.58 (0.46–0.67) to 0.77 (0.66–0.85, *p* = 0.003).

#### 1.1.3. Reinforcement learning for medical imaging

Defining the region of interest (ROI) is often the first step in most image-based radiomics pipelines. This is the case because full patient scans often include artifacts and other information which are irrelevant and can affect the final outcome of the study. Therefore, most pipelines propose a manual localization of a ROI as a preprocessing step. However, it is crucial to define the ROI in an automated and reproducible fashion in other to achieve a fully automated pipeline. We propose a reinforcement learning approach for the localization of the region of interest due to increased speed and lower training data requirements compared to other supervised learning approaches.

Reinforcement learning (RL) has become one of the most active research areas in machine learning and involves the training of a machine learning agent to make a sequence of reward-based decisions toward the achievement of a goal through interaction with the environment. The idea of RL has been long applied in the field of robotics for robot vision and navigation (21–23) before the topic became very popular in the image processing society. RL has been used in the general field of computer vision mainly for object detection (24–26), image segmentation (27, 28), and image enhancement (29–31). However, in medical imaging RL is still in the research phase with very high potential. Netto et al. (32) presented an overview of medical imaging applications applying reinforcement learning with a detailed illustration of a use case involving lung nodules classification which showed promising results. Sahba et al. (27) implemented an RL based thresholding for segmenting prostate in ultrasound images with results that showed high potential for applying RL in medical image segmentation. Alansary et al. (33) evaluated reinforcement learning agents for anatomical landmark detection by comparing fixed and multi-scale search strategies with hierarchical action steps in a coarse-to-fine manner and achieved a performance better than state-of-the-art supervised learning methods.

### 1.2. Main contributions

In this study, we employ parametric information (*T*_*max*_, rBF, rBV) from MR perfusion images of patients with acute ischaemic stroke and predict the three-level DSA based grading of these patients based on this functional information. We hypothesize that the rich information on blood flow visible from MRI perfusion can be used to predict collateral flow in a similar manner to DSA. Moreover, we argue that this approach, using 3D information, may even offer a more reliable biomarker than the interpretation of DSA images. As collateralization patterns are often unstable and may undergo significant changes in the course of minutes, a second estimate of the activation of collateral flow using MRI—in addition to the subsequent DSA—will offer better diagnostic information.

We explore machine learning and deep learning methods in collateral flow grading. We apply deep reinforcement learning, a variant of RL which combines the power of deep learning and reinforcement learning, to detect a rigid-sized cube around the occluded region in an acute ischemic stroke patient scan as an initial step toward the prediction of cerebral collateral flow grading. This step is necessary to automate the detection of the occluded region which improves the accuracy of the prediction. Reducing the time spent on this task and ensuring that the proposed methodology is fully automated.

We provide experiments on different feature extraction strategies including denoising autoencoder (DAE), histogram of oriented gradient (HOG), and local binary pattern (LBP). The extracted features are further utilized in a random forest (RF), K-nearest neighbor (KNN), support vector machine (SVM), and convolutional neural network (CNN) classifiers for the prediction of the collateral flow grading. We provide detailed experimental setup and results which will serve as a guide for further research in this direction.

## 2. Methodology

**Figure 1:**
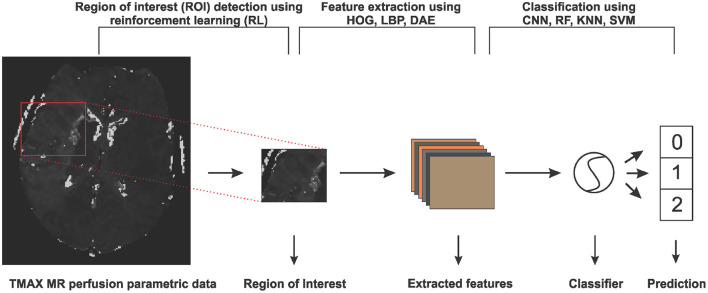
An overview of the steps involved in predicting collateral flow grading from MR perfusion parametric data. The first step involves a region of interest detection using reinforcement learning, followed by histogram of gradient (HOG), local binary pattern (LBP), and denoising autoencoder (DEA) feature extraction schemes and then the classification step which uses random forest (RF), K-nearest neighbor (KNN), support vector machine (SVM), and convolutional neural network (CNN) classifiers.

In this section, we will discuss the details of the steps we employed in predicting the collateral flow grading from MR perfusion data. Figure 1 shows an overview of the main steps involved in the classification process. The first step is the detection of the region of interest (ROI) using reinforcement learning. This step helps to narrow down the classification task to only the area which has been occluded from normal blood flow. The second step deals with extracting features from the ROI. Finally, we feed the extracted features to a set of classifiers to obtain the collateral flow grading for the given patient data.

### 2.1. Deep reinforcement learning for region of interest detection

The idea of reinforcement learning includes an artificial agent which is trained to interact with an environment through a sequence of reward-based decisions toward a specific goal. At every time step *t*, the agent takes into account its current state *s* and performs an action *a* in a set of actions *A* and receives a reward *r* which is a measure of how good or bad the action *a* is toward the achievement of the set goal. The aim of the agent, which is to find an optimal policy (set of states, actions, and rewards) that maximizes the future reward, can be formulated as a Markov Decision Process. Since Markov Decision Process involves a large number of possible decision points which are normally not fully observable, RL approximates the optimal decision function by iteratively sampling from the set of policies through a process known as Q-learning.

#### 2.1.1. Q-learning

At time point *t* and state *s*, let $\pi ={a_{i}}_{i=t}^{t+T}$ be a policy that is a sequence of actions needed by the agent to move from the current state *s* to the target. Let *Q*_*t*_ be a future discounted reward function such that


(1) $$\begin{array}{l} Q_{t}\left(s,\pi \right)=\overset{t+T}{\underset{i=t}{\sum }}\gamma^{i-t}r_{\pi i}, \end{array}$$

where *r*_π*i*_ is the reward associated with the action *a*_*i*_ of policy π at time *t* = *i*, γ∈[0, 1] is the future reward discounting factor, and *T* is the number of steps needed to reach the target by the chosen policy π. At any time step *t* the optimal policy π^*^ is the policy that maximizes the expected value of *Q*_*t*_. This can be represented by an action-value function $Q_{t}^{*}\left(s\right)$ defined by


(2) $$\begin{array}{l} \pi^{*}=Q_{t}^{*}\left(s\right)=\underset{\pi }{\max}𝔼\left[Q_{t}\left(s,\pi \right)\right] \end{array}$$

The optimal value function $Q_{t}^{*}\left(s\right)$ obeys the Bellman equation, stating that if the optimal value $Q_{t+1}^{*}\left(s\right)$ of the next state is known for all possible policies π, then the optimal behavior is to select the policy π^*^ that maximizes the expected value of *r*_π*t*_+*Q*_*t*+1_(*s*, π) [which follows from setting *i* = *t* in Equation (1)]. The action-value function can therefore be estimated recursively as


(3) $$\begin{array}{l} Q_{t}^{*}\left(s\right)=\underset{\pi }{\max}𝔼\left[r_{\pi t}+Q_{t+1}\left(s,\pi \right)\right] \end{array}$$

If the problem space is small enough then the set of policies and state can be fully observed and Equation (3) can be used to determine the optimal policy toward the target. However, in most cases, the problem space is too complex to explore, and hence evaluating the future reward for all possible policies is not feasible. $Q_{t}^{*}\left(s\right)$ is therefore approximated by a non-linear deep network Q^*^(*s*, θ) with a set of parameters θ resulting in what is known as deep Q-learning (34).

#### 2.1.2. Agent state, action definition, and reward function

Given a 3-D scan as the agent's environment, a state *s* is represented by (*s*_*x*_, *s*_*y*_, *s*_*z*_) which is the top-left corner of a (64 × 64 × 64) cube contained in the 3-D scan. We adopt an agent history approach which involves feeding the last four states visited by the agent to the network to prevent the agent from getting stuck in a loop. Since we have a fixed-sized cube as a state our agent's set of six actions {*m*_*u*_, *m*_*d*_, *m*_*l*_, *m*_*r*_, *m*_*f*_, *m*_*b*_} is made up of only movements up, down, left, right, forward, and backward respectively which enables the agent to visit all possible locations within the volume. The agent's reward for taking an action *a* is a function of the intersection over union (IoU) of the target state s^*^ and the state before (*s*_*ab*_), and after (*s*_*aa*_) taking the action. This is given by


(4) $$\begin{array}{l} R_{a}\left(s_{aa},s_{ab}\right)=sign\left[IoU\left(s_{aa},s^{*}\right)-IoU\left(s_{ab},s^{*}\right)\right] \end{array}$$

where *sign* is the sign function that returns −1 for all values less than 0 and 1 otherwise. This leads to a binary reward (*r*∈{−1, 1}) scheme which represents good and bad decisions respectively. During the training stage, the agent search sequence is terminated when the IoU of the current agent's state and the target state is greater than or equal to a predefined threshold τ. At test time the agent is terminated when a sequence of decisions leads to an oscillation [as proposed by Alansary et al. (33)], that is when the agent visits one state back and forth for a period of time.

Experiments by Alansary et al. (33) and Navarro et al. (35) show that deep reinforcement learning has superior performance in object detection as compared to classical supervised learning, especially in images with a noisy background. RL agents also require lesser training data as compared to other supervised learning methods like CNN. These proven advantages make deep reinforcement learning the right choice for our limited and noisy data.

### 2.2. Feature extraction and classification

Feature extraction methods are used in many machine learning tasks to either reduce the dimension of the problem or to extract information from the raw input which would otherwise not be easily extracted by the underlying classifier. In this work, we extract two main classes of features—learned features through a denoising auto-encoder (DAE), and local image descriptors made up of histogram of oriented gradients (HOG) and local binary pattern (LBP).

#### 2.2.1. Denoising auto-encoder

An auto-encoder is an unsupervised deep learning method used for dimension reduction, feature extraction, image reconstruction or denoising and is sometimes also used as a pre-training strategy in supervised learning networks. An auto-encoder is made up of two parts: an encoder Φ:X→F which maps an image *x*∈*X* to $f_{x}\in F$ in the features domain and a decoder Ψ:F→X which maps a feature set f∈F to *x*_*f*_∈*X*. The full auto-encoder is therefore a composite function of the form Ψ•Φ:X→X. Let $\hat{y}=\text{Ψ}\left(\Phi \left(x\right)\right)$ for a given input image x∈X, then the learning process of auto-encoder involves finding a pair of {Φ, Ψ} such that ${\hat{y}}_{i}=x_{i}$ for all $x_{i}\in X$. The encoder Φ then becomes the feature extractor which is used for extracting the needed features.

**Figure 2:**
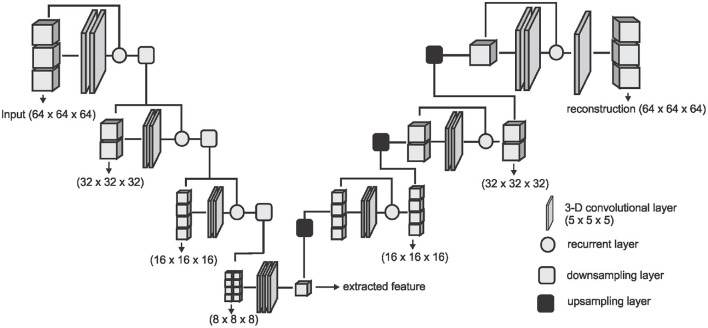
The network architecture used for extracting the DAE features. The downsampling layer is a convolution with a stride of (2 × 2 × 2) which downsamples the input volume to half of the size on every axes. The upsampling layer is a transposed convolution with a stride of (2 × 2 × 2) which doubles the size of the input on every axes.

If the function Φ is invertible, then the learning process can lead to a trivial solution by just choosing Ψ to be the inverse of Φ, and Ψ°Φ becomes an identity function leading to what is known as identity-function risk. To prevent this, the input image *x* is first corrupted by adding noise before feeding it to Φ leading to a denoising auto-encoder. We therefore have


(5) $$\begin{array}{l} \hat{y}=\text{Ψ}\left(\Phi \left(\tilde{x}\right)\right),\;\tilde{x}=\Upsilon \left(x\right) \end{array}$$

where Υ is the random image corruption function. We approximate the encoder and decoder by deep CNNs *E*(*x*, θ_*e*_) and *D*(*f*, θ_*d*_) parameterized by θ_*e*_ and θ_*d*_; respectively. Training is done through back-propagating the Mean Squared Error (MSE) of the original image *x* and the reconstructed image $\hat{y}$ given by


(6) $$\begin{array}{l} L=\frac{1}{N}\overset{N}{\underset{i=1}{\sum }}{\left({\hat{y}}_{i}-x_{i}\right)}^{2} \end{array}$$

where *N* is the number of images in the training set or training batch. We adopt the V-Net architecture proposed by Milletari et al. (36) and simplify it by removing the fine-grained feature forwarding, and reducing the depth of the network due to limitations on the amount of training data available. The downsampling layers of the VNET architecture represent the encoding part [*E*(*x*, θ_*e*_)] of the DAE and the upsampling layers represent the decoding part [*D*(*f*, θ_*d*_)] of the DAE. Figure 2 shows an overview of the simplified architecture used for extracting the DAE features.

#### 2.2.2. Local image descriptors and classifiers

We consider two types of local image descriptors - histograms of oriented gradients (HOG) and a local binary pattern (LBP). Given a volume *X*, we extract the LBP encoding of each voxel by thresholding its 3 × 3 × 3 neighborhood by the intensity value p^*^ of the center voxel which results in 26 long bits *b*_0_, *b*_1_, *b*_2_,..., *b*_25_ where $b_{i}=\left\{1,\text{if}\;p_{i}\geq p^{*},0\;\text{otherwise}\right\}$ and *p*_*i*_ is the intensity value of the *i*th neighbor. We then concatenate the binary encoding to a single binary number *b*_0_*b*_1_*b*_2_...*b*_25_ and then into a decimal value which results in 2^25^ possible binary codes. Details of the implementation until this point can be found in Heikklä and Pietikäinen (37). We group the codes into two main classes—uniform codes which have at most two binary transitions and non-uniform codes which have more than two binary transitions. A binary transition is a switch from 0 to 1 or vice versa. For example the codes 0000, 000111, 011100, and 110110 have zero, one, two, and three transitions respectively. To handle noisy data and to reduce the feature space, we group all the non-uniform codes into one class and add it to the uniform codes resulting in 927 codes instead of 2^25^ . Finally, the histogram distribution of the individual codes is extracted as the LBP feature representation for the volume *X*.

We also explore the HOG feature extractor based on the method proposed in Klas¨er et al. (38). Given a volume *X*, we quantize gradient orientations over an icosahedron and merge opposite directions in one bin resulting in 10 gradient orientations. The gradient for each voxel *x*_*i*_∈*X* is obtained by convolving the 5 × 5 × 5 neighborhood of the voxel by gradient filters *k*_*x*_, *k*_*y*_, and *k*_*z*_ of the same size, giving us a gradient vector ${\vec{x}}_{i}\in {\mathbb{R}}^{3}$. The gradient filters are zero everywhere except for the center columns along the respective axes *k*_*x*_(*i*, 3, 3) = *k*_*y*_(3, *i*, 3) = *k*_*z*_(3, 3, *i*) = [1, 0, −2, 0, 1] for *i*∈{1, 2,..., 5}. The gradient vectors ${\vec{x}}_{i}$ are then projected to the gradient orientations and a histogram representation of these orientations are obtained and used as the HOG feature representation of the volume *X*.

**Figure 3:**
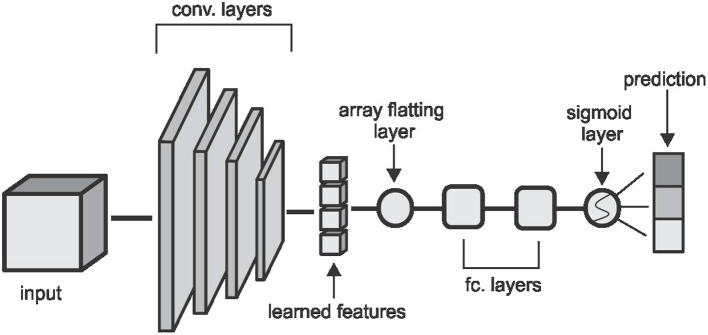
The CNN architecture used in the classification task. Convolutional layers are made up of (5 × 5 × 5) kernels with a stride of (2 × 2 × 2) which reduces the volume by half of the input size on each layer. The first two layers extract 2 feature cubes and the last two layers extract 4 feature cubes each. The fully connected layers have 64 and 32 hidden nodes respectively and the convolutional and fully connected layers are followed by a non-linear hyperbolic tangent (tanh) activation function.

We run experiments with four machine learning classifiers on each of the features extracted. We implement Convolutional Neural Network (CNN), Random Forest (RF), Support Vector Machine (SVM), and K-Nearest Neighbor (KNN) classifiers. Our CNN classifier in Figure 3 has four convolutional layers, aimed at extracting local image features, followed by two fully connected layers and a sigmoid layer for classification. Each layer is followed by a non-linear hyperbolic tangent (tanh) activation function. For classification based on the HOG, LBP, and DEA features, we remove the convolutional layers and feed the features directly to the fully connected layers and then the sigmoid layer for the classification. For the RF, SVM, and KNN classifiers we use the implementation of these classifiers from the Scikit-Learn library (39) in python.

## 3. Experiments and results

### 3.1. Patient population and image data

We test our proposed methods on parametric volumes extracted from MR perfusion data from 183 patients with acute ischemic stroke. Details of the image acquisition and preparation are already published by Pinto et al. (40). Our dataset is made up of three parametric information—*T*_*max*_ volumes which refer to the time taken for the blood flow to reach its peak, relative blood flow (rBF) volumes which refer to the volume of blood passing through a given brain tissue per unit of time, and relative blood volume (rBV) defined as the volume of blood in a given brain tissue relative to an internal control (e.g., normal white matter or an arterial input function). Each volume has a resolution of (0.9, 0.9, and 6.5 mm) and a dimension of (256, 256, and 19) voxels on the sagittal, coronal and axial planes respectively. Ground truth labels are obtained from a trained neuroradiologist, with over ten years of experience, who manually investigates the DSA slides of the associated patient and assigns one of three labels (0-poor, 1-medium, 2-good) to this patient. We use these labels for a 3-class prediction experiment and we also experiment on a risk-stratified nested test where we first predict good - (2) against not good (0, 1) collaterals and then separate the not good class into poor (0) and medium (1) collaterals in a cascaded approach.

### 3.2. Preprocessing

Our image preprocessing involves two main tasks. First, we make our datasets isotropic by applying a B-spline interpolation to the axial axis since the other two axes have the same spacing leading to volume with a resolution of 0.9 mm on each plane and a new dimension of (256, 256, and 127). This is followed by an extraction of the brain region from the skull using the brain extraction tool (BET) from the ANTS library. The brain extraction is carried out on the *T*_*max*_ volumes and the resulting mask is then applied to the rBF and rBV volumes.

### 3.3. Region of interest localization

**Figure 4:**
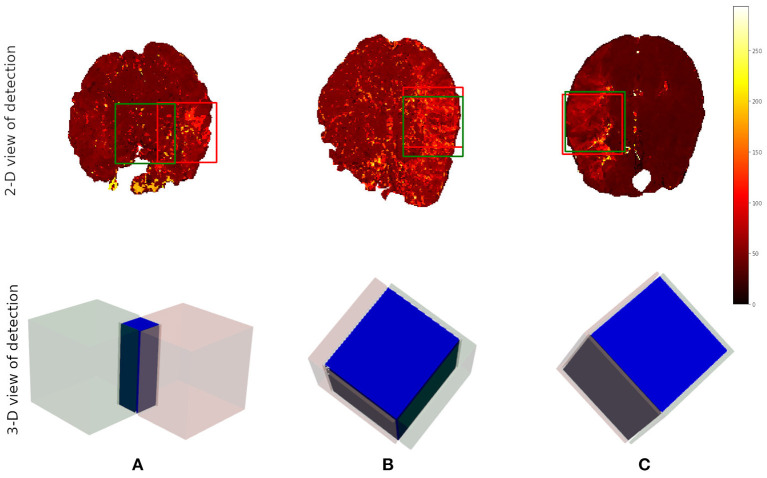
Qualitative results from the ROI detection task. The top row is the axial view of the ground truth (in red) and the prediction (in green). The bottom row is a 3-D visualization of the ground truth cube (in red), the predicted cube (in green), and the intersection between the two (in blue). Column **(A)** corresponds to the worst prediction in our test set while column **(C)** refers to the best result in terms of IoU. In column **(B)**, we can observe that though the overlap is not perfect the prediction still contains some part of the occluded region which is not in the ground truth. This implies that though we have poor scores we still have good ROI detection which can be used for the classification task.

**Figure 5:**
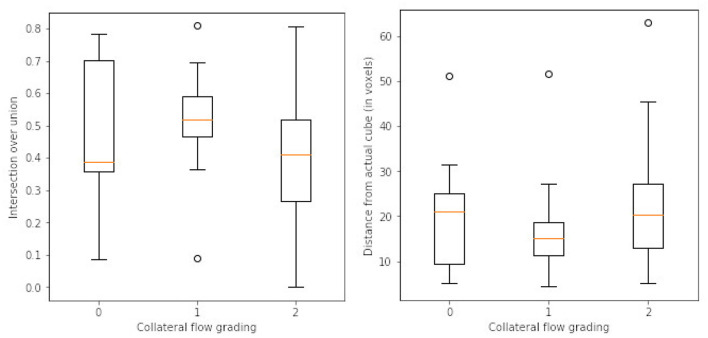
Box plots of results from ROI detection task. **Left** is the intersection over union (IoU) ratio between the prediction and the ground truth over the three classes. **Right** is the euclidean distance between the predicted center point and the ground truth center point. From the distributions, it is clear that it is easy to detect the ROI in the poor collateral flow class (class 0) compared to the good collateral flow class (class 2). This can be explained by the fact that in good collateral flow cases *T*_*max*_ shows uniform values in the whole volume.

After the preprocessing step we extract the occluded regions as the region of interest (ROI) using the reinforcement learning architecture described in Section 2.1. We adopt the network architecture from Alansary et al. (33) with modifications proposed in Navarro et al. (35). A stopping criterion of τ = 0.85 is used during training—that is, an intersection over union (IoU) value greater than or equal to 0.85 implies that the region of interest is detected. We perform the ROI detection task on the *T*_*max*_ volumes since the occluded regions are easier to detect in these volumes. The resulting cube region is then applied on the rBF and rBV volumes to extract the corresponding cubes in these volumes as well. For each volume, we select 20 starting cubes of size (64 × 64 × 64) at random and run the agent till the stopping criterion is reached. We then aggregate the results from the 20 different runs to get the prediction of the final ROI. After getting the region we extract the mirror of the ROI (ROI+M) by reflecting the ROI on the opposite side of the brain and using it as an additional feature. This results in 6 cubes per patient (i.e., two volumes each from *T*_*max*_, rBF, and rBV volumes). Qualitative and quantitative results from the region of interest extraction can be found in Table 1 and Figures 4, 5. From the box plots in Figure 5, it is evident that the region of interest detection was more successful in the poor collateral flow classes (class 0 and 1) than in the good collateral flow class. This can be explained by the fact that in cases of good collateral flow, there is a uniform distribution of the *T*_*max*_ value within the occluded region and its neighborhood making it hard for the RL agent to detect the ROI. From Figure 4 we observe that in most cases the ground truth does not cover the total occluded region [e.g., column (b)] and hence the predicted ROI, though does not completely overlap the ground truth, still contains other parts of the occluded region which is not captured in the ground truth and it is therefore sufficiently accurate for the classification task.

**Table 1:** Quantitative results from the region of interest detection task.

**Type**	**Class**	**Mean**	**Std**	**Max**	**Min**
IoU	0	0.49	0.22	0.79	0.08
	1	0.52	0.14	0.81	0.09
	2	0.42	0.21	0.81	0.04
Center points displacement (in voxels)	0	20	13	51	5
	1	17	9	52	4
	2	23	14	63	5

IoU refers to the intersection over union ratio between the prediction and the ground truth. Center point displacement is the euclidean distance between the predicted center point and the ground truth center point.

### 3.4. Classification

#### 3.4.1. Feature representations

In total three sets of features (DAE, HOG, and LBP) are extracted in addition to the actual extracted cube (ROI) and its mirror cube (ROI+M). We learn features automatically through an unsupervised denoising auto-encoder. The network takes the extracted ROI cubes from the *T*_*max*_, rBV, and rBF volumes as three input channels and produces a single channel feature set of size (8 × 8 × 8). We normalize the cubes individually into the range [0, 1] before feeding them to the network.

**Figure 6:**
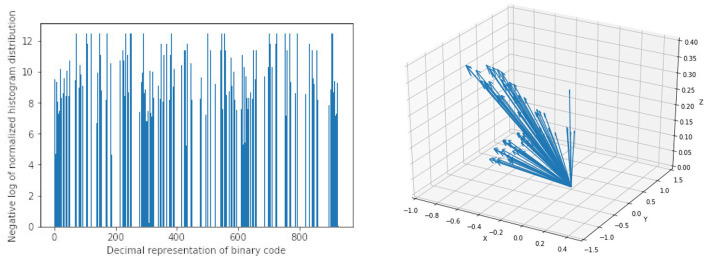
Sample of extracted features using Local Binary Pattern (LBP) on the **left** and Histogram of Oriented Gradients (HOG) on the **right**. Bar heights from LBP features represent the frequency of a given pattern and the position on the x-axis is the decimal representation of the binary pattern. Arrow directions in HOG features are the gradient vectors and the length of the arrows represents the frequency of the given gradient. The LBP features show a uniform distribution of the extracted patterns with no dominant pattern. The HOG features on the other hand show evidence of high gradients in the extracted region of interest.

For HOG features we extract 10 features each for the three parametric volumes and concatenate them into a vector of length 30 for the classification task. Figure 6 shows a sample of the extracted HOG features for a patient for the three input channels. Finally, LBP features are extracted using the method described in Section 2.2. Here we combine all the three channels and run the histogram over the three channels which results in a 927 feature vector as explained in Section 2.2. Figure 6 shows a sample of the extracted LBP features from our dataset.

#### 3.4.2. Classifier training

We handle the collateral flow classification through two main approaches—a three-class multi-label classification task where we predict three labels in one step, and a two-step cascaded approach where we predict a binary label of classes (0, 1) against 2 in the first step and separate the class 0 from 1 in the second step. We implement our CNN architecture using the Keras library (41) in python with TensorFlow as the backend. Random forest, support vector machine, and K-nearest neighbor classifiers were implemented using the scikit-learn library (39) in python. We set up our experiments as follows:

***CNN classifier***: For the CNN classifier, we use a weighted categorical cross-entropy with a weight of $\frac{1}{\left|k\right|}$ for each class *k* in the training set. A stochastic gradient descent optimizer with a learning rate of 0.001, decay of 1*e*^−6^, and momentum of 0.9 is used to fine-tune the network parameters at 20 epochs.

***K-Nearest neighbor classifier***: We conduct preliminary a experiment with a grid search to know which parameters will work best. For our final experiment, we use *k* = 3 neighbors with uniform weights, a leaf size of 30, and the Minkowski metric.

***Random forest classifier***: After the initial grid search experiment, we implement the classifier with 200 estimators, and the Gini impurity function is used to measure the quality of a split.

***Support vector machine classifier***: We use a regularization parameter *C* = 10, a third-degree polynomial kernel, a balanced class weight, and a tolerance of 1*e*^−3^ for the stopping criterion.

#### 3.4.3. Classification results

We test different combinations of the feature sets extracted in the previous experiments and classifiers discussed in a preliminary experiment and present the results in Table 2. Due to limitations in the size of the dataset, we adopt a 5-fold cross-validation approach in a preliminary experiment instead of a training-validation-test splitting approach and report the average scores over the accuracy in the individual validations. In the preliminary experiments (results in Table 2), we use the manually annotated ROI and not the ROI predicted from the proposed reinforcement learning. We later, in a follow-up experiment, compare the performance of the proposed CNN on manually annotated ROI and the predicted ROI (results in Table 3).

**Table 2:** Results from preliminary experiments on collateral flow grading.

**Type**	**Method**	**RAW**	**ROI**	**ROI+M**	**DAE**	**HOG**	**LBP**
Three classes	CNN+MLP	0.51(±0.04)	0.63(±0.06)	0.65(±0.03)	0.50(±0.07)	0.38(±0.13)	0.25(±0.14)
	RF	0.51(±0.02)	0.65(±0.04)	0.67(±0.05)	0.66(±0.04)	0.69(±0.02)	0.60(±0.05)
	KNN	0.48(±0.10)	0.54(±0.02)	0.58(±0.05)	0.55(±0.06)	0.59(±0.02)	0.43(±0.04)
	SVM	0.56(±0.04)	0.66(±0.05)	**0.70**(±**0.03**)	0.70(±0.04)	0.53(±0.02)	0.25(±0.15)
Cascaded (two step)	CNN+MLP	0.55(±0.01)	**0.72**(±**0.05**)	0.70(±0.04)	0.66(±0.05)	0.54(±0.02)	0.21(±0.13)
	RF	0.47(±0.07)	0.67(±0.03)	0.64(±0.03)	0.65(±0.04)	0.70(±0.03)	0.56(±0.07)
	KNN	0.44(±0.04)	0.55(±0.06)	0.56(±0.07)	0.52(±0.08)	0.60(±0.05)	0.48(±0.08)
	SVM	0.38(±0.02)	0.51(±0.04)	0.46(±0.04)	0.51(±0.04)	0.46(±0.04)	0.10(±0.00)

RAW features refer to the full-sized three parametric volumes (*T*_*max*_ , rBF, and rBV) after skullstripping. ROI refers to the corresponding cubes extracted from the parametric volumes based on the manually annotated ROI and ROI+M is the ROI combined with its mirror cube on the opposite side of the brain. Other features (DEA, HOG, and LBP) are all extracted from the ROI cubes. Scores represent mean accuracy over the 5-fold cross-validation experiments with their corresponding standard deviation in parenthesis.

Values in bold refer to the feature-classifier combination with the highest accuracy under each experiment type.

**Table 3:** Results from the experiment on collateral flow grading using only ROI data on our proposed cascaded CNN.

**Input data**	**Binary**	**Three classes**
Manual ROI	0.84	0.74
Automated ROI	0.80	0.72

Manual ROI refers to the ground truth ROI and automated ROI refers to the predicted ROI from our proposed Reinforcement Learning approach. Binary refers to the result from the first binary classification (i.e., {0,1} vs. 2) and three classes is the three-class classification based on the cascaded networks.

The results in Table 2 show that the region of interest extraction step helps improve the results in all classification methods. This can be verified by comparing the performance from the full image (RAW column) with the performance of the region of interest (ROI column) in Table 2. Also by adding the mirror of the occluded region to the extracted ROI (ROI+M) we achieve improved results in most of the classifiers with performance falling in classifiers like KNN and SVM due to the increase in the dimension of data introduced by the mirror of the ROI. The cascaded method shows higher accuracy in almost all the classifier-feature combinations when compared to the direct three-class prediction. This can be explained by the distribution of classes in the dataset. That is, for the cascaded approach we have fairly balanced data when we combine poor and moderate flow against good collateral flow which is not the case with the direct three-class multi-label prediction approach. It, therefore, suggests that in cases where we have highly imbalanced class distributions a multi-label classification might perform poorly. The overall performance of CNN is better than the other machine learning classifiers and can be explained by the fact that the convolutional layers of the CNN architecture extract features while paying attention to the class of the input data. This makes the feature extraction process more efficient than the other feature extraction schemes which have no knowledge of the underlying label of the input data at the time of extracting the features. Again CNN with only ROI data performs slightly better than with the mirror of the ROI (72 vs. 70% in Table 2) and this can also be explained by the fact that the CNN used in our experiments is fairly shallow and hence could not handle the additional feature dimensions introduced by the mirrored images.

Based on the results of the preliminary experiment, we further probe into the training of the proposed CNN classifier with the ROI data. In this experiment, we split the data into training and testing sets. The test set is made up of 50 volumes randomly selected with reference to the ratio of class count in the entire dataset. We make use of both the manually annotated ROI and the automated ROI from our proposed Reinforcement Learning approach during training. We finally evaluate the trained models on the automated ROI and compare it with the same network trained and evaluated solely on the manually annotated ROI data. Table 3 shows the result of this experiment.

The results in Table 3 from our follow-up experiment show that the automated ROI from the proposed Reinforcement Learning approach is comparable to the manually detected ROI in terms of predicting collateral flow (2% drop in accuracy which represents one out of the 50 patients in the test set). This is crucial in automating the whole collateral flow prediction workflow in a clinical setting.

## 4. Summary and conclusion

In this work, we present a deep learning approach toward grading collateral flow in ischemic stroke patients based on parametric information extracted from MR perfusion data. We start by extracting regions of interest using deep reinforcement learning. We then learn denoising auto-encoder features and modern implementation of 3-D HOG and LBP features. We proceed to the actual classification task using a combination of the extracted features and CNN, random forest, K-nearest neighbor, and support vector machine classifiers.

Our experiments show that the rich information on blood flow visible from MRI perfusion can be used to predict collateral flow in a similar manner to DSA images which are invasive in nature. Region of interest detection with reinforcement learning is successful to an acceptable level and can be used as a guide to estimate the region in the brain which requires more attention. It is evident that high class imbalance can be a major challenge in the collateral flow grading task and many similar works. We however show that for datasets with high class imbalance, a two-step cascaded classification approach performs better than a one-time multi-label classification method. It is also evident from our results that a direct CNN classifier is able to extract relevant features from the region of interest and has an advantage over classical machine learning classifiers like RF, KNN, and SVM that depend on handcrafted features like HOG and LBP.

Collateral flow grading is an essential clinical procedure in the treatment of ischemic stroke patients. We have presented a framework for automating the process in clinical setup and have achieved promising results given our limited dataset. For the proposed framework to be clinically useful there is the need for further tests with possibly more data from multiple stroke centers. The grading can also be customized for specific patient groups for example providing information about age group, gender, and other biographical and historical information of patients as an additional feature can help improve the result of the framework.

## Data availability statement

Data is currently private, pending approval from the source of the data. Requests to access these datasets should be directed to giles.tetteh@tum.de.

## Author contributions

GT and BM contributed to the conception and design of the study. GT, FN, and JP contributed to the experiments and computer codes. RM, JK, and RW organized the database. GT wrote the first draft of the manuscript. BM, JP, and JK contributed to the final manuscript. BM, CZ, JK, and RW handled the administrative tasks. All authors contributed to manuscript revision, read, and approved the submitted version.
